# Emerging Role of isomiRs in Cancer: State of the Art and Recent Advances

**DOI:** 10.3390/genes12091447

**Published:** 2021-09-20

**Authors:** Veronica Zelli, Chiara Compagnoni, Roberta Capelli, Alessandra Corrente, Jessica Cornice, Davide Vecchiotti, Monica Di Padova, Francesca Zazzeroni, Edoardo Alesse, Alessandra Tessitore

**Affiliations:** 1 Department of Biotechnological and Applied Clinical Sciences, University of L’Aquila, Via Vetoio, 67100 L’Aquila, Italy; veronica.zelli@univaq.it (V.Z.); chiara.compagnoni@univaq.it (C.C.); roberta.capelli@graduate.univaq.it (R.C.); alessandra.corrente@graduate.univaq.it (A.C.); jessica.cornice@graduate.univaq.it (J.C.); davide.vecchiotti@univaq.it (D.V.); monica.dipadova@univaq.it (M.D.P.); francesca.zazzeroni@univaq.it (F.Z.); edoardo.alesse@univaq.it (E.A.); 2 Center for Molecular Diagnostics and Advanced Therapies, University of L’Aquila, Via Petrini, 67100 L’Aquila, Italy

**Keywords:** isomiRs, miRNome, miRNA variants, expression pattern, cancer

## Abstract

The advent of Next Generation Sequencing technologies brought with it the discovery of several microRNA (miRNA) variants of heterogeneous lengths and/or sequences. Initially ascribed to sequencing errors/artifacts, these isoforms, named isomiRs, are now considered non-canonical variants that originate from physiological processes affecting the canonical miRNA biogenesis. To date, accurate IsomiRs abundance, biological activity, and functions are not completely understood; however, the study of isomiR biology is an area of great interest due to their high frequency in the human miRNome, their putative functions in cooperating with the canonical miRNAs, and potential for exhibiting novel functional roles. The discovery of isomiRs highlighted the complexity of the small RNA transcriptional landscape in several diseases, including cancer. In this field, the study of isomiRs could provide further insights into the miRNA biology and its implication in oncogenesis, possibly providing putative new cancer diagnostic, prognostic, and predictive biomarkers as well. In this review, a comprehensive overview of the state of research on isomiRs in different cancer types, including the most common tumors such as breast cancer, colorectal cancer, melanoma, and prostate cancer, as well as in the less frequent tumors, as for example brain tumors and hematological malignancies, will be summarized and discussed.

## 1. Introduction

MicroRNAs (miRNAs) are small, non-coding RNA molecules able to negatively regulate the expression of target genes at the post-transcriptional level. The first miRNA, named lin-4, was discovered in 1993 in *Caenorhabditis elegans* [1]; afterward, in *C. elegans* again, *let-7* was described [2]. Later, miRNAs were identified in both invertebrates and vertebrates. Numerous studies thereafter, that focused on unveiling the role of these molecules, highlighted the importance of miRNAs functions in the regulation of fundamental biological processes, such as cell proliferation, growth, apoptosis, and metabolism. Today, more than 1900 annotated precursors are listed in the miRbase database for *Homo sapiens* (www.mirbase.org, accessed on 20 June 2021). Based on gene annotation, miRNAs show different genomic locations in mammals: in *H. sapiens*, 52%, 40%, and 8% appear to reside in intergenic, intronic, or exonic regions, respectively [3]. Dysregulation of miRNAs’ expression was widely described, gaining high attention, in several human pathological conditions that are of a high impact on public health, such as cardiovascular disease [4,5], neurological disease [6], and cancer [7]. Regarding the latter, depending on the fine-tuning role in the post-transcriptional regulation of tumor suppressors or oncogenes, miRNAs have been classified as oncomiRs or tumor suppressor miRs [8]. Furthermore, microRNAs are considered as potentially suitable tissue or circulating non-invasive diagnostic, prognostic, and predictive biomarkers in several pathological conditions, including cancer [9,10]. With this regard, miRNAs are thought to be released from tissues by active (exosomes, vesicles) or passive (apoptosis, necrosis) mechanisms into the bloodstream, where they are stable and resistant to endogenous RNase. The stability of miRNA in body fluids can be ascribed to macromolecular complex formation, including association with AGO2 or HDL [11,12,13], and/or microvesicle/exosome encapsulation [14,15,16,17]. Furthermore, the possible use and delivery of miRNAs as therapeutic targets is under investigation [18,19].

Next-generation sequencing (NGS) technology has revolutionized and significantly improved the approach to molecular analyses. The great potential of NGS technology is the ability to massively sequence millions of reads, allowing the simultaneous detection of many variants and different gene expression patterns, using a very low amount of nucleic acids with considerable time and cost reduction [20]. With the advent of NGS technologies, several miRNA variant sequences were detected, initially ascribed to sequencing errors/artifacts [21,22]. However, based on the calculated sequencing error rate, they were encountered more frequently than would be expected [23]. This observation led to the hypothesis that such non-canonical variants in length and sequence, now named isomiRs, originated from physiological processes that affect canonical miRNA biogenesis [24,25,26]. IsomiRs biological activity and functions are not fully understood, but, given their frequency in the human miRNome and their putative functions in cooperating with the canonical miRNAs or exerting different roles as well [27,28], they are a subject of interest for additional new analyses. Moreover, a study based on bioinformatics data elaboration, revealed that annotation, batch variable (i.e., sequencing platform, plate, sample quality, and sequencing depth) correction and quality control of The Cancer Genome Atlas (TCGA) datasets could help to better estimate the real isomiRs frequency, with a consequently better understanding of their putative biological role in cancer [29]. In this scenario, it is undeniable that further analyses are needed to clarify the role of isomiRs, given not only their potentially relevant role in human diseases but also their putative use as biomarkers. In this review, we summarize the current knowledge about the involvement and possible functions of isomiRs in several types of cancer.

## 2. From the Canonical microRNAs to isomiRs’ Discovery

MicroRNAs are short, 19–24 nucleotides in length, non-coding RNA molecules playing a role in negatively regulating translation either by inhibition or target mRNA degradation. In the canonical pathway, pri-miRNAs are produced in the nucleus by Polymerase II. Afterward, the RNase III Drosha, with the microprocessor complex subunit DGCR8 (DiGeorge critical region 8) cleaves pri-miRNAs, thus generating 60–100 nucleotides in length hairpin precursors (pre-miRNAs) which are transferred to the cytoplasm by Exportin 5. There, pre-miRNAs are cleaved again by the RNase III Dicer, giving rise to mature double-stranded miRNAs which associate to a member of Argonaute (AGO1-2-3-4 paralogs) family proteins to produce the miRISC (miRNA-induced silencing complex), with one strand preserved (guide) and the other one degraded (passenger). MiRNAs effects are principally induced by the interaction between the seed region (nucleotides 2–8 at the microRNA 5′ end) and specific partially or perfectly complementary microRNA responsive elements (MREs) in the target mRNA, mainly located at the 3′-UTR, leading to translation inhibition or mRNA degradation, respectively [30,31]. Complex and not completely understood processes are at the base of the different modalities of gene expression silencing which involve, among other factors, AGO members [32,33,34,35]. Even though more in-depth and clarifying analyses are needed, previous studies demonstrated an essential role of AGOs in endonucleolytic cleavage, translation repression, or mRNA destabilization. AGO2 was shown to possess slicer activity, whereas AGO1-3-4 were described to have mainly a slicer-independent activity and to play a role in deadenylation and translation repression [36,37,38]. It is known that a single miRNA can potentially interact with hundreds of target genes and that multiple miRNAs can regulate the same mRNA [39,40,41]. With this regard, to assist, predict, and assess the complicated network of miRNAs:mRNAs interactions, several bioinformatics approaches [42], algorithms and tools have been designed [43,44].

Usually, microRNAs are characterized by specific sequences. However, in 2007, Landgraf et al. [45] sequenced over 250 small RNA libraries from 26 different organ systems and cell types from humans and rodents and described miRNA variants in approximately 20% of clone sequences. Similarly, in 2008, Morin et al. [24] observed that miRNAs from human embryonic stem cells frequently showed sequence variations from their canonical form, coining the term isomiR to refer to these multiple variants.

These sequence variations were initially ascribed to post-transcriptional modifications or PCR/sequencing technical errors, but, with the advent of NGS technologies coupled with more sophisticated analysis algorithms, it was demonstrated that such newly discovered variants cannot be considered as technical artifacts, but rather attributable to molecules with in vivo functional and evolutionary importance [28,46].

## 3. isomiRs’ Classification

**Figure 1:**
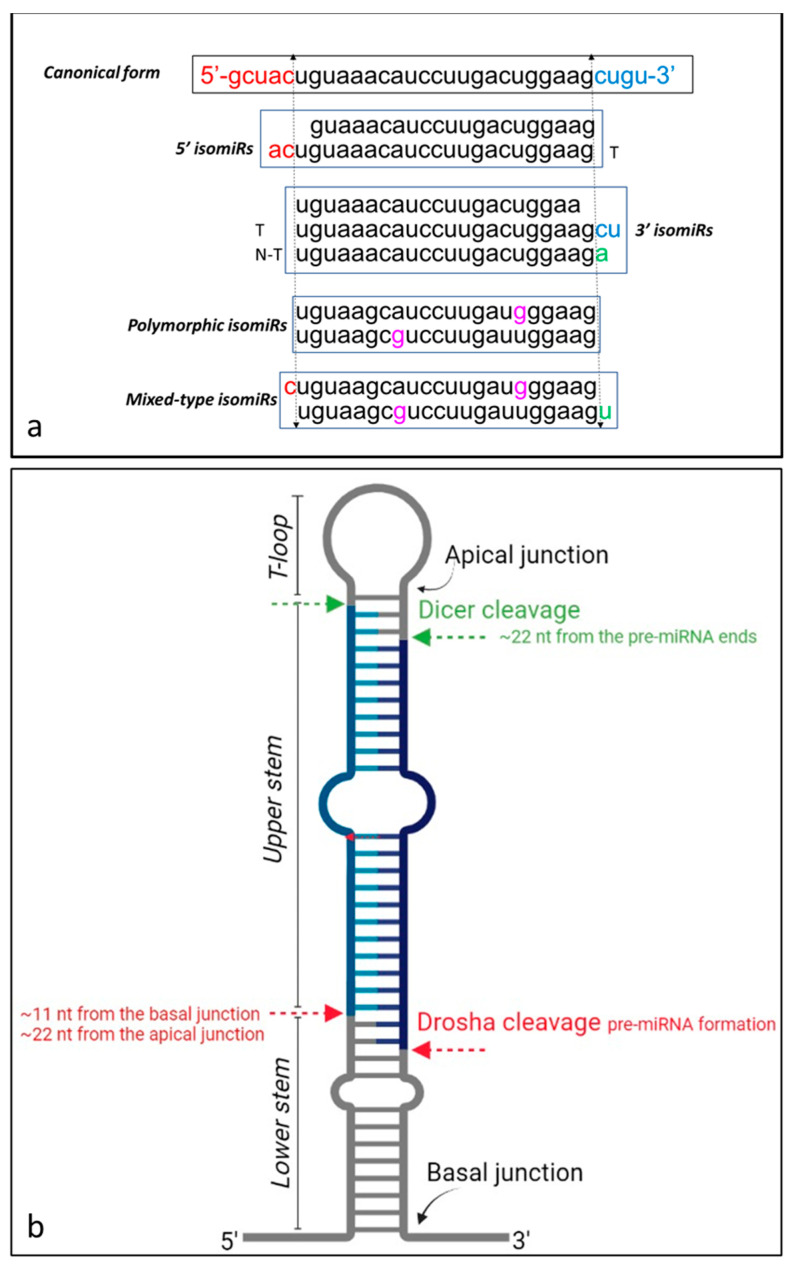
(**a**) Schematic of isomiRs classification and related modifications compared to the canonical form (5′ isomiR, 3′ isomiR, polymorphic and mixed-type isomiRs) (hsa-miR-30e-5p sequence as an example). T: template, N-T: non-template, based on the correspondence of the added nucleotides with the flanking precursor sequence; (**b**) miRNAs and possible isomiRs biogenesis through sequential cleavages mediated by Drosha and Dicer enzymes (image generated by using BioRender https://biorender.com/ accessed on 20 June 2021).

IsomiRs are heterogeneous molecules characterized by different nucleotide sequences and/or lengths with respect to their canonical counterparts. As reported by Wu et al. [47], besides the canonical forms and depending on the modification, four classes of isomiRs are identified which include: (1) isomiRs with changes at the 5′ end; (2) isomiRs with changes at the 3′ end; (3) isomiRs showing polymorphisms within the sequence, without any difference in length; (4) isomiRs with polymorphic plus 5′ and/or 3′ changes (mixed type). Extensive classification also identifies three classes for 5′ and 3′ isomiRs which are mutually exclusive: with deletion/addition, both referred to nucleotides at 5′ or 3′ end, and with variations, referred to changes at the level of 5′ or 3′ nucleotides, including extensions of the original length. Moreover, the addition of nucleotides can be further defined as template or non-template, depending on the correspondence of the added nucleotides with the flanking precursor sequence (Figure 1a).

## 4. Biogenesis of isomiRs

IsomiRs can arise from different and not completely understood mechanisms; below the most important are reported.

### 4.1. Drosha/Dicer Non-Canonical Cleavage

In the canonical biogenesis pathway, the miRNA 5′ and 3′ termini are generated by sequential cleavages of the primary transcript mediated by Drosha and Dicer enzymes [48]. Drosha/DGCR8 complex induces a cleavage at the level of specific positions with respect to the basal (approximately 11 nucleotides away) and the apical (approximately 22 nucleotides away) junctions of the pri-miRNA; Subsequently, Dicer recognizes the pre-miRNA termini via the PAZ domain and cuts the short hairpin pre-miRNA in a site located approximately 22 nucleotides away from the 5′/3′ ends, thus removing the terminal loop and generating the duplex miRNA/miRNA* containing guide and passenger strands [49] (Figure 1b). Experimental models suggest that Drosha cleavage imprecision appears related to modification of the pri-miR loop size, or to alterations of the stem length or the single-stranded sequences at its base. Similarly, Dicer cleavage imprecision seems to be dependent on pre-miR structure and sequence (reviewed by Tomasello et al. [26]). In this scenario, cleavage imprecision can generate new 5′ and 3′ variants with possibly different functions [50,51,52]. Isoforms varying in length can be ascribed, other than to imprecise cleavage, to the removal of the end(s), or to the addition of nucleotides: in the first and second case, template conditions are generated, whereas in the third one a non-template condition generally occurs [25]. Bofill-De Ros et al. [53] analyzed the maturation of three paralogs of pri-miR-9 (pri-miR-9-1/2/3), demonstrating that pri-miR-9-1 possessed a unique Drosha cleavage attributable to its flexible and distorted stem structure. After cleavage, pri-miR-9-1 was described as the only paralog able to generate an alternative *miR-9* with a shifted seed sequence which showed a potential role in low-grade glioma progression.

Both genetic and epigenetic alterations in miRNA processing proteins can affect miRNA biogenesis and expression. For example, germline and somatic mutations in *DROSHA*, *DGCR8*, *DICER1*, and *XPO5* genes have been identified in several cancers, including Wilms tumors [54,55], pleuro-pulmonary blastoma [56], non-epithelial ovarian cancer [57], endometrial, colon, and gastric tumors [58]. Since some of the processing proteins involved in miRNA biogenesis are also implicated in the generation of miRNA variants, the presence of alterations in these genes could represent another mechanism responsible for the isomiRs generation and different expression levels in several diseases, including cancer. Interestingly, in a recent study, Galka-Marciniak et al. [59] analyzed cancer somatic mutations in miRNA biogenesis genes and suggested that *DICER1* hotspot mutations could impact the expression levels of some isomiRs.

### 4.2. Exoribonucleases and Nucleotidyl Transferases

In *Drosophila melanogaster*, the 3′-to-5′ Nibbler exoribonuclease was able to induce 3′ end trimming in more than 25% of miRNAs of *Drosophila*, after AGO1 loading. Trimming of miRNA 3′ ends may occur as a final step in RISC assembly and is likely required to enhance target mRNA repression. *Nibbler* depletion caused the formation of miRNAs intermediates longer than 22 nucleotides. On a phenotypic level, this led to developmental defects as Nibbler is necessary for normal fly development of *D. melanogaster*, indicating a relevant physiological function [60] and encouraging further explorations in higher organisms.

Nucleotidyl transferases, mainly with uridyl or adenosyltransferase activity, are defined as template-independent polymerases able to add nucleotides at the 3′-terminus of RNAs. The nucleotide additions impact miRNA stability and performance, are conserved in animals, occur at certain loci, and are catalyzed by enzymes involved also in the regulation of other RNAs, suggesting the importance and specificity of their role [61,62,63]. PAPD4/5, ZCCHC6/11 in humans, TUT1, MTPAP, and PAPOLG are the most relevant nucleotidyl transferases described in isomiRs’ biogenesis [61,64,65,66].

### 4.3. Editing of RNA and SNPs

Adenosine-to-inosine (A-to-I) is the most frequent (20%) modification in the pre-miR sequence. It is mediated by the adenosine deaminases ADARs, principally ADAR1/2 [67,68]. A-to-I change can occur in the processing of pri-miRNA and pre-miRNA; if within the seed sequence, given the identification of I as G, RNA editing can affect the role of the modified molecules, resulting for example in the regulation of new target genes [69,70].

Single nucleotide Polymorphisms (SNPs) occur at a 1% frequency in the human genome and are involved in determining the inter-individual diversity and susceptibility to diseases. SNPs in miRNAs have been associated with defects in miRNAs maturation as well as alterations in miRNA:mRNA interactions in cancer.

For example, the alteration in the biogenesis of the tumor suppressor *miR16-1* and its association with the increased risk of familial chronic lymphocytic leukemia was reported by Calin et al. [71] and attributed to mutations on the pri-miR-15a/16-1.

Similarly, the SNP rs2910164 in the pre-miRNA of *miR-146a* has been linked to the risk of developing cervical cancer [72], while an SNP in the pri-miRNA of *miR-128b* has been associated with acute lymphocytic leukemia [73].

Several databases (e.g., PolymiRTS, miRNASNP) listing genetic variants in miRNAs’ seed regions are now available [74,75], and the effects of some variants have been demonstrated in several pathological conditions [76,77,78,79].

## 5. Significance of isomiRs’ Functions

The aforementioned alterations of miRNAs molecules that lead to isomiRs’ generation are suggestive of modifications of their functional capability. Overall, isomiRs activity can be correlated to miRNAs’ affected stability and/or target selection and seems to play a role either in supporting [46] or competing [80] with their canonical counterparts. Regarding targeting efficiency, most isomiRs show 3′ variations [28], leaving the 5′ end and the seed region unmodified, with subsequent proper target detection. However, it has been described that the 3′-terminus plays a role to reinforce and maintain stability and specificity during miRNA-MRE interaction. This has been demonstrated especially in presence of mismatches within the seed region [81,82,83], indicating also that 3′ modifications could have an impact on functions of such miRNA isoforms. On the other hand, variations (additions or deletions) at the level of the 5′ end may cause a seed shifting with consequent functional effects [84]. This can generate the gain or loss of functions in terms of target recognition [85].

In the miRISC, microRNAs guide AGO proteins at the sites of target mRNA 3′ UTR, thus inducing repression of translation or mRNA degradation, depending on the seed region complementarity [86,87]. Four paralogs of structurally similar AGO proteins (AGO1-2-3-4) are expressed in *H. sapiens*. AGO2 was thought to be the only AGO member with RNA slicer activity [88], however, more recently, the same function was ascribed, under particular conditions, to AGO 3 [89]. Many human miRNAs may associate with all AGOs, although several of them are preferably loaded into specific AGO proteins [86]. Several studies highlighted the interaction between isomiRs and AGO proteins, further indicating their functional role [27,90]. Deep-sequencing analysis revealed that some miRNA modifications appear to determine the preference to a different AGO protein loading [91], indicating that isomiRs might affect miRISC function.

### 5.1. isomiRs Detection and Quantification

Several methods can be used for the detection and quantification of isomiRs expression; these primarily include qRT-PCR and NGS approaches.

Poly(A) and stem-loop qRT-PCR are the most common techniques for the analysis of small RNAs due to their high level of specificity and the relatively low impact in terms of experimental times and costs [92]. However, the main limitations are represented by the need to know the sequence, with the consequent impossibility of discovering new molecules, as well as the limited accuracy in the discrimination of sequences that differ by only one/few nucleotides, especially if poorly represented in the sample, as often occurs for miRNA isoforms [93,94].

Alternatively, other PCR-based methods for the analysis of isomiRs expression, include, Dumbbell-PCR [95], two-tailed RT-qPCR [96] as well as Multiplex Single Base Primer Extension Assay [97].

To date, NGS represents the methodology of choice for the detection and quantification of miRNAs and miRNAs variants, particularly for the discovery of new isoforms.

The most important limitation of using this technique is the lack of standardized data analysis protocols and normalization methods, which are essential for obtaining high-quality and reproducible results [47].

Overall, several methods, mainly based on sequence complementarity to precursor, location, and pattern of modified nucleotides or pre-defined isomiRs as well as machine learning approaches were used, depending on research interests or contexts, to explore isomiRs expression patterns [47,98]. NGS offers a great opportunity to specifically and sensitively detect isomiRs. However, the analysis of isomiRs remains a challenge, due to the limited number of specific tools available to analyze their expression levels in sequencing experiments and the lack of comparison among them [47] as well as the possible overestimation of isomiRs detected [99].

### 5.2. Resources and Tools for isomiRs’ Analysis

In 2016, Zhang et al. [100] produced a database listing 308,919 isomiRs from 4706 mature miRNAs (accessible at https://mcg.ustc.edu.cn/bsc/isomir/, accessed on 20 June 2021). Bhattacharya et al. [74] created the PolymiRTS database, with the aim of describing the polymorphisms in microRNAs and their target sites (https://compbio.uthsc.edu/miRSNP/, accessed on 20 June 2021). miRNASNP-v3 is another database for SNPs in miRNAs and target sites (http://bioinfo.life.hust.edu.cn/miRNASNP/, accessed on 20 June 2021) [101]. Very recently, the Tumor Isomir Encyclopedia (TIE, https://isomir.ccr.cancer.gov/, accessed on 20 June 2021) database was created [102], to query and compare isomiR expression across more than 11,000 tumor samples from The Cancer Genome Atlas (TCGA). Several resources for the analysis of isomiRs can be further accessed at https://tools4mirs.org/software/isomirs_identification/ (accessed on 20 June 2021).

## 6. isomiRs in Cancer

IsomiRs can differ from miRNAs in abundance, stability, and role, as they can be functionally redundant or discrepant compared to their canonical counterparts.

It has been proven that isomiRs are constitutively generated, but, while some isoforms are ubiquitous in expression, others exhibit tissue-specific expression and abundance [103]. Thus, the study of isomiRs in cancer could provide further insights into the miRNA’s biology and their implication in oncogenesis.

Increasing evidence suggests that isomiRs likely play a role in the pathogenesis and progression of different cancer types and could also contribute to cancer molecular heterogeneity. A further element of interest is the promising use of isomiRs as cancer diagnostic and prognostic biomarkers; for example, Wang et al. [98] demonstrated the capability of a panel of fifty 5′ end isomiRs to effectively classify more than thirty different tumor types. Moreover, isomiRs expression was proven to be superior compared to miRNAs expression in the classification of these different cancer types [103]. Similarly, Lan et al. [104] showed that breast cancer subtypes classification, based on isomiRs expression, was more effective than gene expression profiles.

In the next sections, the state of research on isomiRs in different cancer types will be discussed. The studies discussed herein, with particular focus on isomiRs identified as unique (tissue-specific) or, in some cases, shared among diverse tumors, their expression level and role, are summarized in Table 1.

### 6.1. Breast Cancer

IsomiRs in breast cancer (BC) were studied for the first time by Chang et al. [123] who used NGS data from the NCBI Sequence Read Archive (SRA) to perform differential expression analysis of miRNAs and isomiRs in tumor and adjacent breast normal tissues. In this study, high frequency of position shift was observed at the 3′ end of miRNAs (accounting for about 40% of all miRNAs reads) and more than 12% of miRNAs reads showed 3′ end nucleotide modifications in samples; however, no significant differences in isomiRs position shifts and 3′ end modifications between tumor and normal tissue were observed.

Similarly, by using NGS data from The Cancer Genome Atlas (TCGA), Wu et al. [124] performed a comprehensive analysis of miRNAs and isomiRs expression in 683 BC and 87 normal tissues. Although one or more expressed isomiRs were detected for each miRNA locus, none of the isomiRs identified showed variations in the seed sequence at the 5′-end position, which is responsible for the interaction between microRNA and target mRNAs. Thus, the authors suggested a possible functional redundancy, due to the shared target genes between isomiRs and their canonical counterparts, with a consequent contribution in regulating the same pathways. Moreover, no statistically significant differences in isomiR expression profiles emerged between BC and normal tissues.

Starting from miR-seq datasets from TCGA, Zhang et al. [105] developed a bioinformatics approach to characterize and compare the isomiR pattern in normal and breast tumor tissues. Interestingly, a total of 71 isomiRs, most of which arising from the same miRNA locus, showed significant differential expression in normal compared to cancer tissues. Functional annotation and pathway-based analysis of the putative isomiRs targets revealed enrichment for genes involved in well-known cancer-related pathways, such as MAPK signaling and focal adhesion.

A bioinformatics pipeline for the analysis of small RNAs, specifically aimed at distinguishing canonical miRNAs and their variants, was also developed by Muller et al. [125], and used for the analysis of primary normal and triple-negative BC (TNBC) cells. By using this tool, the authors identified more than 1000 isomiRs: the majority of them were “templated isomiRs”, most probably deriving from imprecise enzymatic cleavage by Drosha or Dicer leading to variants that perfectly matched the pre-miRNA sequence. IsomiRs that originate from the addition of nucleotides, particularly 3′ end adenylation and uridylation, were also observed both in normal and cancer cells. Interestingly, for 11 miRNAs, including *miR-148b-3p*, *miR-152-3p*, and *miR-23b-3p*, the authors observed that the canonical form appeared poorly or not expressed. On the contrary, the 3′ end non-templated isoform resulted in highly expressed, representing the predominant variant. Overall, results from this study highlighted the importance of improving the methodologies and bioinformatics pipelines for the analysis of miRNAs and their variants, thus analyzing not only miRNAs but also isomiRs expression.

Telonis et al. [106] examined the isomiRs expression profiles by using BC datasets from TCGA. They described not only that isomiRs expression patterns could better distinguish tumor from normal breast tissue than miRNAs patterns, but also that isomiRs were able to discriminate and characterize, at the molecular level, luminal A and luminal B subtypes compared to transcriptome expression profiles. Differential expression of several isomiRs, such as variants from TJU CMC MD2.ID00121 (a novel human-specific miRNA locus), *miR-125a* and *miR-183-5p*, was also correlated to patients’ ethnicity/race; for example, a statistically significant over-expression of different variants of *miR-183-5p* was observed in triple-negative BC (TNBC) cases from Caucasian patients, but not from African-Americans. Furthermore, three different isoforms of this miRNA demonstrated a different impact on transcriptome profile in MDA-MB-231 BC cells.

A study on the expression pattern and interaction between coding and non-coding RNAs in normal and TNBC patients, again in Caucasian vs African-American women, was also performed by Telonis et al. [107], in order to analyze the regulatory effect of non-coding RNAs on transcriptome profile and to shed light on race-specific molecular differences. By adding evidence to their previous study [106], the authors identified differentially expressed isomiRs in normal and tumor tissues: in particular, numerous variants of *mir-21-5p*, *mir-182-5p*, and *mir-183-5p* were found to be up-regulated, while several isoforms of *miR-10b* and *miR-99a* (both 3p and 5p) were found down-regulated in TNBC compared to normal samples. Furthermore, isomiRs, including variants arising from *miR-200c*, *miR-21*, the *miR-17/92* and the *miR-183/96/182* clusters, were associated with race-related disparities in terms of metastases-correlated pathways, since a co-expression of these isomiRs and genes belonging to the Wnt signaling pathway was observed almost exclusively in African-American TNBC patients. Of note, the canonical form of some isomiRs here identified (e.g., *miR-21-5p*, *miR-200* and *miR-10* families and the *miR-183/96/182 cluster*) included miRNAs known to be altered in BC.

TNBC tumors were also analyzed by Guo et al. [126], who performed small RNA profiling in 26 TNBC cell lines and compared the expression levels of non-coding RNAs among the four intrinsic TNBC subtypes [basal-like (BL1, BL2), mesenchymal (M), and luminal androgen receptor (LAR)]. They identified several differentially expressed small RNAs (including isomiRs) among the different TNBC subtypes, none of which, however, was unique to each TNBC subtype compared to all other subgroups.

Salem et al. [108] reported high expression levels of shifted 5′ end isoform of *miR-140-3p*, a potential tumor suppressor miRNA previously associated with stemness regulation in BC, both in BC cell lines and tumors. Functional characterization revealed that overexpression of 5′ isomiR-140-3p, but not of *miR-140-3p*, led to decreased cell viability and migration in BC cell lines, and induced cell cycle arrest. Due to the 5′ end alteration and the resulting shift of the seed sequence, a different spectrum of target genes was expected. Indeed, genes involved in cell viability, migratory potential, and cell cycle regulation (*COL4A1*, *ITGA6*, and *MARCKSL1*), emerged as three novel specific target genes of the 5′ isomiR-140-3p. Overall, the authors suggested a functional synergy, not a functional redundancy, between 5′ isomiR-140-3p and its canonical miRNA, highlighting that the tumor suppressor role of the above-reported two small RNAs may be regulated by different molecular mechanisms.

In a cell line model of early-stage TNBC, according to Salem et al. [108], Bhardwaj et al. [109] identified the 5′ isomiR of *miR-140-3p* (miR-140-3p-1) and the new targets HMG-CoA reductase (*HMGCR*) and HMG-CoA synthase 1(*HMGCS1*), two key genes involved in the cholesterol biosynthesis during the multi-step tumorigenic process, as central regulators of TNBC development. Decreased expression of both isomiR and canonical miRNA during cancer progression (from non-cancer parental cell line to invasive cancer) was observed; indeed, miR-140-3p-1 acted as a tumor suppressor, with loss of miR-140-3p-1 promoting upregulation of *HMGCR* and *HMGCS1*.

Lan et al. [104] explored for the first time the potential role of isomiRs as biomarkers for the identification of BC subtypes. By using novel bioinformatics and machine learning methods, they demonstrated the efficacy of isomiRs in distinguishing different BC subtypes, also improving the knowledge of the molecular mechanisms underlying BC heterogeneity. Twenty isomiRs were identified as key biomarkers for BC subtype classification. Pathway-based analysis of the predicted target mRNAs revealed five pathways that were significantly affected by isomiRs and known to be involved in BC, notably that of p53, MAPK and Estrogen signaling.

To deepen the relationship between altered miRNAs expression and epithelial-to-mesenchymal transition (EMT) in BC, Rhodes et al. [110] analyzed and demonstrated the suppressive effect of *miR-200b-3p* and *miR-200b-5p* on EMT in TNBC cell lines. Interestingly, they observed that ectopic expression of pri-miR-200b led to the formation of *miR-200b-3p* and *miR-200b-5p* as well as multiple isoforms of both miRNAs, characterized, in particular, by heterogeneity at 3′ position. However, the possible role of these variants in the context of EMT has not been further investigated.

Finally, Koi et al. [111] observed higher expression levels of a specific 3′ isomiR of *miR-21-5p*, together with *miR-23a-3p* and tRF-Lys (TTT), in serum from BC patients compared to healthy controls, also demonstrating the high accuracy of a model combining these 3 small RNAs as a putatively useful diagnostic tool for the detection of early-stage BCs. *MiR-21-5p* is a known oncogenic miRNA, involved in cancer proliferation and invasion. Of note, altered expression levels of the same 3′ isomiR of *miR-21-5p* identified in this study (3′ addition C) were also detected in tissues from colon cancer patients [47], suggesting that this isomiR could be involved in the pathogenesis of different types of tumors.

### 6.2. Other Common Tumors: Colorectal Cancer, Melanoma, Prostate Cancer

Despite the limited number of studies, several proofs indicate that isomiRs generation and their higher expression levels compared to the canonical forms, represent a widespread phenomenon in common tumors, including colorectal cancer, melanoma, and prostate cancer.

#### 6.2.1. Colorectal Cancer

Wu et al. [47] developed a sequence-oriented isomiRs annotation (CASMIR) method to specifically analyze isomiRs expression profiles from small-RNA sequencing data, which was tested in colorectal cancers (CRCs), advanced adenoma, and normal samples. IsomiRs were identified as the most abundant small RNAs in CRC samples, accounting for about 70% of total miRNA reads. Among them, the expression levels of isomiRs of *miR-21-5p* (3′ addition C), *miR-27a-3p* (3′ deletion C), *miR-30e-5p* (3′ addition CU), *miR-125a-5p* (3′ deletion GA) and *miR-224-5p* (3′ addition U) were found 7 to 18-fold higher than their respective canonical counterparts. Of note, for *miR-21-5p*, a widely studied miRNA upregulated in CRC, the greatest abundance of 3′ addition C form suggested a more discriminating role than the canonical form. Fifty-eight isomiRs, including isoforms of *miR-135b-5p*, *−182-5p*, *−183-5p*, *−192-5p*, *−200b-3p*, *−96-5p*, *−200a-3p*, *−200c-3p*, and *−429*, were also found up-regulated in both CRCs and advanced adenoma compared to normal tissues. Overall, the over-expressed isomiRs identified in this study belong to miRNAs families known to be involved in oncogeneses, such as *miR-17-92, miR-200*, and *miR-183* families.

Mjelle et al. [112] identified a large fraction of differentially expressed miRNAs and isomiRs in colon cancer compared to normal samples as well as in microsatellite instability (MSI) positive and negative tumors. Interestingly, they observed that about 50% of detected miRNAs showed higher variants’ expression compared to their canonical forms; overall, 2451 isomiRs, arising from 343 unique miRNAs, were found differentially expressed in tumor and normal samples. Among MSI-positive and negative tumors, *miR-26a-5p* isomiRs appeared differentially expressed in 48 patients analyzed by sequencing in this study and in TCGA datasets as well.

#### 6.2.2. Melanoma

Throughout the study of miRNA expression profiling in melanoma, Kozubek et al. [127] described the presence of widespread non-coding small RNAs, including isomiRs, highlighting the complexity of melanoma transcriptome and miRNome landscape. Among the different miRNA isoforms, miR-451a.1, arising from *miR-144/451a* cluster, emerged as the most abundant isomiR of *miR-451a*.

The same was extensively studied by Babapoor et al. [113], which demonstrated its association with amelanotic phenotype and tumor-suppressive effects, by inhibition of melanoma invasion/progression. MiR-451a.1 expression was increased in normal skin, with a progressive decrease in melanoma and dysplastic nevi compared to common nevi. After ectopic expression of *miR-144/451a* in melanoma cell lines, higher levels of mature miR-451a.1 with respect to *miR451a* or *miR-144*, and significant reduction of cell migration, as well as inhibition of invasion, were observed.

Through small-RNome analysis of single and multiple primary melanomas (PMs), Dika et al. [114] identified a set of 17 isomiRs, mainly belonging to miRNA families involved in tumorigenesis, including *miR-200, miR-30, and miR-10* families, that showed higher expression than their canonical forms. In particular, the miR-125a-5p|0|-2 isoform was 10-fold more abundant than the canonical counterpart and showed differential expression levels in the first tumor with respect to the second one in patients with multiple PMs. Target prediction analysis revealed functional modification of the miR-125a-5p|0|-2 isoform and target gene pattern change, leading to the inability of this isomiR to target key genes involved in cell adhesion and migration (*Ephrin receptors*, *Netrin 1*) or intracellular signaling (*PIK3C2B*).

Londin et al. [115] performed small RNA-sequencing in 80 primary uveal melanomas, the subtype arising from melanocytes of the uveal tract, and identified a complex regulatory network involving several small RNAs, including isomiRs. The authors observed that the majority of the identified isomiRs derived from best-studied miRNA loci, such as *miR-21-5p*, *miR-183-5p*, *miR-143-3p*. Increased or decreased expression levels of isomiRs from multiple miRNA loci, some of which previously correlated to pathways involved in tumor development and melanoma proliferation, were also associated with molecular phenotypes, disease progression, and patient survival: for example, increased levels of miR-21-5p|−1|0 and miR-29a-3p|−1|1 and decreased levels of miR-99a-3p|1|1 and let-7c-5p|−1|1 were observed in patients with metastases.

#### 6.2.3. Prostate Cancer

Magee et al. [128] studied isomiRs and tRNA-derived fragments (tRFs) in a large cohort of prostate cancer (PC) samples from the TCGA dataset. Numerous isomiRs were identified, many of which arose from few miRNA precursors, including *miR-143-3p*, *miR-182-5p*, *miR-21-5p*, *miR183-5p*, *miR-10a-5p*, and *let-7a-5p*. Like that previously reported in BC [80], they found different isomiRs expression levels related to patients’ ethnicity.

In order to evaluate the potential role of non-coding RNAs as biomarkers of radiation response in PC, Leung et al. [129] explored the possible impact of radiation on miRNA levels, arm selection preference, and isomiRs in a model of radiation-treated PC3 cells. Regarding isomiRs expression, the authors observed altered 3′ modification of miRNAs after radiation exposure. A gradual increase in the proportion of dinucleotide modifications at the end of the miRNA reads was also observed in PC3 cells after radiation treatment.

Circulating miRNAs and isomiRs, detectable in biofluids such as serum or urine, are emerging as very promising non-invasive biomarkers for disease states. Koppers-Lalic et al. [116] investigated the expression pattern of miRNAs in urine extracellular vesicles (EVs) of PC patients compared to healthy controls. Interestingly, significant differential expression was detected for isomiRs of *miR-21*, *miR-204*, and *miR-375*. A model to evaluate the diagnostic performance of these 3 isomiRs also demonstrated a higher AUC (area under the curve) value than prostate-specific antigen (PSA), highlighting the promising potential of this panel of 3 isomiRs as a diagnostic tool in PC.

### 6.3. Other Cancer Types

A limited number of papers have been published about isomiRs detection, frequency, and function in other, less common, cancer types. In the present section, a summary of the literature available on the role of isomiRs in several cancers and cancer cell lines including brain tumors, hematological malignancies, gastrointestinal (GI) tract cancers, thyroid carcinoma, and laryngeal cancer is provided.

IsomiRs from *miR-9* and *miR-219*, two microRNAs involved in neuronal development and differentiation, were found specifically expressed in low-grade glioma, suggesting a potential role as biomarkers in this type of tumor [103].

*MiR-210-3p* and related isomiRs emerged as high hypoxia-inducible small RNAs in glioblastoma cell lines. The authors showed that several additional isomiRs, mainly differing from the canonical forms for a few nucleotides mostly at the 3′ end, also displayed differential expression profiles under hypoxia [117].

Kuchenbauer et al. [50] described isomiRs in a murine leukemia progression model. The authors observed that almost all detected miRNAs presented as one or more variants, identifying a total of 3390 isomiRs from 225 different miRNAs. Although the potential role of these variants in post-transcriptional regulation was not investigated, the authors highlighted that the miRNA transcriptome landscape was more complex than previously supposed.

Guo et al. [130] hypothesized that small RNA alteration levels could be implicated in the transformation of myelodysplastic syndromes (MDS) to Acute Myeloid Leukemia (AML). Besides five miRNAs, including three members of the *miR-181* family, they identified two differentially expressed isomiRs (miR-10a-5p+1 and hsa-miR-92a-3p+2) in MDS patients who never progressed to AML compared with MDS patients who progressed to AML; however, the difference of the two isomiRs’ expression was not statistically significant after adjustment for blast percentage and age.

The expression and function of isomiRs from the *miR-27* family in cancer were analyzed by Ma et al. [118]. Through the use of miRNAs and isomiRs transfection into AML cells (AML-12 cells), they demonstrated the suppressive role of canonical *miR-27b* on the expression of metabolism-related proteins, including PEPCK, G6Pase, FAS, and CPT1A. However, this inhibitory effect was considerably decreased when AML-12 cells were transfected with isomiR-27b-1 or isomiR-27b-2, suggesting the possible regulatory function of such isomiRs in biological processes, not necessarily redundant with respect to their canonical form.

By RNA-sequencing analysis of T-cell acute lymphoblastic leukemia (T-ALL) patients, Wallaert et al. [131] identified 2139 isomiRs arising from 481 different miRNAs: for 106 of them, the canonical form was not detected, suggesting that several miRNAs could often be more represented by alternative forms rather than the canonical form. An association between the expression levels of specific miRNAs and the number of isomiRs arising from them was also observed; an example is represented by *miR-181a-5p*, which showed the highest average expression in T-ALL, as well as 39 different isoforms, detected. Similarly, Dawidowska et al. [132] performed small RNA sequencing of 34 pediatric T-ALL samples, including the characterization of isomiR expression patterns. They observed that the majority of miRNAs (about 70%) detected were represented by up to 10 isoforms.

The small RNA transcriptional landscape of 30 primary multiple myeloma tumors, including the discovery and characterization of isomiRs, was analyzed by Agnelli et al. [133]. Among the isoforms detected, about 76% were characterized by shorter or longer mature species without mismatches. In some cases, the expression of the variants was higher than the canonical form or represented up to the whole signal, as was noted for *miR-150-3p*.

Finally, Loher et al. [134] analyzed isomiR expression profiles in lymphoblastoid cell lines (LCLs) from five different population groups. Several isomiRs, with modifications at the 5′, 3′ end, or both, were identified and showed population- and gender-specific expression patterns. Overall, expression levels of 76 isomiRs showed statistically significant population-dependent differences; among them, the authors focused on variants from *miR-1304-3p* (in particular, an isomiR which lacked 2 nucleotides at the 3′-end with respect to the canonical form) and variants from *miR-143-3p* (in particular, an isomiR characterized by an extra nucleotide at the 3′-end). Isoforms from these two miRNAs were found more abundant in Yoruban Africans (both males and females) and European ancestry (only in females) compared with other populations, respectively.

Regarding GI tract cancers, in order to investigate miRNAs/isomiRs and circular RNAs expression profiles as well as possible interactions among them, Guo et al. [135] analyzed tumor and adjacent-normal samples from patients with esophageal cancer. They observed altered expression levels for several miRNAs and isomiRs. Furthermore, the number of predicted miRNA-circular RNAs interactions was significantly increased when isomiRs were considered for the analysis. Overall, the authors highlighted the potential functional relationship and crosstalk complexity of the different deregulated RNA species in esophageal cancer, particularly related to the expression of isomiRs.

The potential role of circulating miRNAs and isomiRs as non-invasive biomarkers in esophageal cancer was investigated by Ibuki et al. [119] through the analysis of esophageal squamous cell carcinoma (ESCC) samples and matched normal controls. A model including *miR-30a-5p* and two isomiRs (variants of *miR-574-3p* and *miR-205-5p*) demonstrated high diagnostic performance, with an AUC value of 0.95 and sensitivity and specificity parameters higher than 80%, highlighting the promising potentiality of this three small RNAs panel as a diagnostic tool in ESCC.

Sequencing analysis of small RNAs, including isomiRs, from a gastric tumor and its corresponding normal tissue, was performed by Li et al. [136]. The authors reported a different 5p/3p arm ratio of the same pre-miRNAs in the two tissue types and differential expression preferences in the enrichment of specific isomiRs, with different variants from the same miRNA which preferentially occurred in normal or gastric tissue, respectively (e.g., *miR-497* and *mir-21*). In other cases, differences in the abundance of the same isomiRs (e.g., *let-7a*) as well as differences in the expression levels of canonical miRNAs, but similar isomiR distribution pattern (e.g., *miR-30b*) were observed, demonstrating a wide and diverse range of isomiRs distribution in normal and tumor tissue.

The analysis of transcriptome and small RNA profiles of extracellular vesicles (EVs) cargoes from four liver-cancer cell lines revealed a shared background as well as cell line-specific characteristics in terms of both coding and non-coding RNA species [137]. Regarding isomiRs expression, miRNAs variants accounted for about 26–30% of the total miRNAs reads across all the four cell lines. As expected, 3′ end isomiRs were the predominant category, although internal nucleotide modifications, alone or in combination with 3′ or 5′ end alterations, were also found. Overall, the authors observed a high correlation between the expression of isomiRs and their canonical counterparts, suggesting a possible similar biological role of miRNAs and corresponding isoforms in EVs context. Indeed, isomiRs can be functionally redundant to their canonical miRNAs, as it was reported for miR-139-5p variants, in particular miR-139-5p -1|-1, for which a synergistic role to suppress tumor development and progression by targeting *IGF1R* was observed in hepatocellular carcinoma [120].

MicroRNA transcriptome in normal thyroid and papillary thyroid carcinoma (PTC) was analyzed by Swierniak et al. [121]. Several tissue-specific isomiRs, including 5′ end isomiRs with alteration of the seed sequences and consequent differences in the putative target genes recognition, were detected. Six different 5′ end isoforms of *miR-146b-5p*, a highly deregulated miRNA in PTC, were shown. These variants, whose expression was 14–29 times higher in tumor than in control tissue, were characterized by the modification of one nucleotide, leading to the formation of two alternative seed regions whose predicted target genes overlapped for only 13%. Similarly, the passenger strand of the pre-miRNA (*miR-146b-3p*) produced four 5′ end isoforms, with the generation of isomiRs with 2 different seed regions; in this case, the predicted target genes which were regulated by both seeds accounted for 9.4%.

By contrast, Saiselet et al. [138] did not observe correlations between isomiRs, in terms of isoforms distribution, 5p-to-3p arm expression ratios and non-templated additions, and PTC tumorigenesis or lymph node metastases process.

Parafioriti et al. [139] performed miRNoma profiling in chondrosarcoma tissues and identified several differentially expressed miRNAs and isomiRs in grade II-III compared with grade I samples. Analyzing predicted target genes, particularly for 5′ end isoforms, the authors observed that many of the pathways targeted by these isomiRs were involved in cancer-associated signaling, also suggesting a synergistic role with the canonical counterparts.

Finally, small RNA-sequencing analysis revealed quantitative and qualitative alterations in the expression of isomiRs of *miR-196a* in laryngeal cancer, with the presence of high levels of isomiRs with complete length in laryngeal cancers, and low levels of mature *miR-196a* as well as expression of a truncated variant (deletion of 1–2 nucleotides at both ends) in normal laryngeal tissues [122].

## 7. Discussion and Conclusions

To date, it is well established that miRNAs can be present in several isoforms that are heterogeneous in length and/or sequence. IsomiRs generation is not a rare event and has been estimated to contribute to half of the miRNome in human cells. The discovery of isomiRs, and their inclusion in miRNome analysis, highlighted the complexity of the small RNA transcriptional landscape in several diseases, including cancer [50].

Recent studies have characterized both the expression and the possible biological function of isomiRs arising from a single miRNA as well as the isomiRs expression patterns within a specific cancer type.

While some isomiRs appear to be tumor-specific, others emerged as ubiquitously expressed in several human cancers and cancer cell lines. For example, high expression levels of isomiRs arising from miRNAs families known to be implicated in human tumorigenesis, particularly *mir-21-5p*, *miR-200*, and *miR-183* families, were frequently observed and shared among almost all cancer types.

However, isomiRs are a recently identified class of molecules and most studies to date are focused on the type and abundance of isomiRs compared to canonical miRNAs rather than on the differential expression analysis in different tumor types and subtypes, or on the analysis of the biological role of altered classes of isomiRs.

In conclusion, data on different cancer types suggest that isomiRs generation is a widespread phenomenon in tumors. Based on the known and growing importance of miRNAs as promising diagnostic and prognostic markers, and the molecular and clinical heterogeneity of cancers as well, further analyses are required to better characterize the expression patterns of isomiRs and their role in cancer pathogenesis and progression to provide new insights into cancer heterogeneity.

## Figures and Tables

**Table 1:** IsomiR identified in different cancer types.

IsomiR(s)	Cancer Type	Expression Level/Described Role	Reference
Panel of 71 isomiRs	Breast Cancer	Differentially expressed in tumor and normal breast tissues	[105]
Isoforms of TJU CMC MD2.ID00121, *miR-125a* and *miR-183-5p*	Breast Cancer	Differentially expressed with regard to BC patient’s race	[106]
Isoforms of *mir-21-5p*, *mir-182-5p*, *mir-183-5p*	Breast Cancer	Up-regulated in TNBC compared to normal samples	[107]
Isoforms of *miR-10b* and *miR-99a*		Down-regulated in TNBC compared to normal samples	
Isoforms of *miR-200c*, miR-21, *miR-17/92* cluster and *miR-183/96/182* cluster		Associated with race disparities in Caucasian and African-American TNBC patients	
5′ isomiR-140-3p	Breast Cancer	Tumor suppressor role via regulation of cell viability, cell migration, and cell cycle	[108]
5′ isomiR-140-3p	Breast Cancer	Tumor suppressor role, involved in TNBC development	[109]
Variants of *miR-200b-3p* and *miR-200b-5p*	Breast Cancer	Observed following ectopic expression of pri-miR-200b in breast cancer cell lines	[110]
Panel of 20 isomiRs	Breast Cancer	Able to distinguish BC subtypes	[104]
3′isomiR of *miR-21-5p* (3′ addition C)	Breast Cancer	Over-expressed in serum of BC patients compared to controls	[111]
*MiR-21-5p* (3′ addition C), *miR-27a-3p* (3′ deletion C), *miR-30e-5p* (3′ addition CU), *miR-125a-5p* (3′ deletion GA) and *miR-224-5p* (3′ addition U)	Colorectal Cancer	Higher expression levels than their canonical counterparts	[47]
Panel of 58 isomiRs (including *miR-135b-5p, −182-5p, −183-5p, −192-5p, −200b-3p, −96-5p, −200a-3p, −200c-3p, and –429* and, overall, *miR-17-92*, *miR-200*, and *miR-183* families.)		Up-regulated in CRC and advanced adenoma compared to normal tissues	
Isoforms of *mir-26a-5p*	Colorectal Cancer	Differentially expressed between MSI positive and negative tumors	[112]
MiR-451a.1	Melanoma	Tumor suppressive role by inhibition of melanoma progression	[113]
Panel of 17 isomiRs (including *miR-200*, *miR-30*, and *miR-10* families)	Melanoma	Higher expression levels than their canonical counterparts	[114]
MiR-125a-5p|0|−2		Higher expression levels than the canonical miRNA and differentially expressed in multiple melanomas in the same patient	
MiR-21-5p|−1|0 and miR-29a-3p|−1|1	Uveal Melanoma	High expression levels associated to metastasis	[115]
MiR-99a-3p|1|1 and let-7c-5p|−1|1		Low expression levels associated to metastasis	
IsomiRs of *miR-21*, *miR-204* and *miR-375*	Prostate Cancer	Putative circulating diagnostic biomarkers	[116]
IsomiRs of *miR-9* and *miR-219*	Glioma	Putative biomarkers in low-grade tumors	[103]
*MiR-210-3p* and related isomiRs	Glioblastoma cell lines	Hypoxia-induced	[117]
IsomiR-27b-1 and isomiR-27b-2	AML-12 cells	Regulation of metabolism-related proteins expression	[118]
Isoform of *miR-574-3p* and *miR-205-5p*	Esophageal Carcinoma	Diagnostic biomarkers	[119]
MiR-139-5p −1|−1	Hepatocellular Carcinoma	Tumor suppressor role by targeting IGF1R	[120]
Isoforms of *miR-146b-5p* and *miR-146b-3p*	Thyroid Carcinoma	Up-regulated in tumors compared to normal samples	[121]
IsomiRs of *miR-196a*	Laryngeal Cancer	Dysregulation in tumor and normal tissues	[122]

Abbreviations: BC, breast cancer, TNBC, triple-negative breast cancer, CTC, colorectal cancer, MSI, microsatellite instability.

## Data Availability

Not applicable.
